# Academic and molecular matrices: A study of the transformations of connective tissue research at the University of Manchester (1947–1996)^☆^

**DOI:** 10.1016/j.shpsc.2010.12.007

**Published:** 2011-06

**Authors:** Miguel García-Sancho

**Affiliations:** Department of Science, Technology and Society, Spanish National Research Council (CSIC), Calle Albasanz, 26-28, 28037 Madrid, Spain

**Keywords:** Connective tissue, Collagen, Manchester, Biomedicine, Rheumatism, Recombinant DNA

## Abstract

This paper explores the different identities adopted by connective tissue research at the University of Manchester during the second half of the 20th century. By looking at the long-term redefinition of a research programme, it sheds new light on the interactions between different and conflicting levels in the study of biomedicine, such as the local and the global, or the medical and the biological. It also addresses the gap in the literature between the first biomedical complexes after World War II and the emergence of biotechnology. Connective tissue research in Manchester emerged as a field focused on new treatments for rheumatic diseases. During the 1950s and 60s, it absorbed a number of laboratory techniques from biology, namely cell culture and electron microscopy. The transformations in scientific policy during the late 70s and the migration of Manchester researchers to the US led them to adopt recombinant DNA methods, which were borrowed from human genetics. This resulted in the emergence of cell matrix biology, a new field which had one of its reference centres in Manchester. The Manchester story shows the potential of detailed and chronologically wide local studies of patterns of work to understand the mechanisms by which new biomedical tools and institutions interact with long-standing problems and existing affiliations.

## 1 Introduction

The investigation of contemporary biomedicine has been a main concern in both historical and contemporary Science and Technology Studies (STS). Historians and sociologists of biology, as well as policy scholars, have investigated the interactions between biologists, physicians, research institutions, clinical sites and funding agencies in the emergence of programmes expected to lead laboratory biology to the diagnosis and treatment of diseases. This has resulted in a wide literature both in scope and approach: the scholarship covers the second half of the 20th century and addresses the development of biomedicine from different perspectives.

One might distinguish between two levels of analysis: micro-studies and wider characterisations of stages and structures in the history of biomedicine. Within the latter approach, historians have investigated the emergence of the first “biomedical complexes” after World War II and their connections with the changing scientific and political priorities in Europe and the United States (Beatty, 1991; Hannaway, 2008; Hardy & Tansey, 2006; Quirke & Gaudillière, 2008). There is also a rich literature on the interactions between laboratories and the pharmaceutical industry, their transformations throughout the 20th century and the development of drugs such as insulin, penicillin or anti-histamines (Bud, 2007; Liebenau, 1987; Liebenau, 1989; Quirke, 2008; Swann, 1988). More recently, historians and policy scholars have addressed the emergence of biotechnology and how the advent of recombinant DNA methods transformed the practice and economics of biomedicine from the 1970s onwards (Collins, 2004; Gottweis, 1998a; Orsenigo, 1989; Wright, 1994). The scholarship on biotechnology is sometimes framed in terms of the emergence of a new form of knowledge production—more interdisciplinary and problem oriented than the previous biomedical complexes—and of a “triple helix” of interactions between the university, governments and industry (Etzkowitz & Leydesdorff, 2000; Gibbons et al., 1994).

The micro-studies address the formation of more specific “platforms” or “networks” oriented towards a disease or research problem (Keating & Cambrosio, 2006). These associations may result in a particular medical innovation, in the emergence of a new discipline or in institutional transformations. Thus, scholars have investigated the connections between laboratories and hospitals in the investigation of cancer or sickle-cell anaemia (De Chadarevian, 1998; Löwy, 1996); and they have explored local configurations of biomedical disciplines such as physiology immunology or molecular biology (De Chadarevian, 2002; Kay, 1993; Tansey, 2008; Tauber & Podolsky, 2000). There are also a number of anthropological studies on the emergence of recent molecular diagnostic tools in laboratories and companies (Rabinow, 1996; Rabinow, 1999; Rabinow & Dahn Cohen, 2005).

All these investigations share a concern with interactions between different and sometimes conflicting levels. A number of studies address national and institutional biomedical traditions, the mobility of researchers and the policy role of institutions such as the Medical Research Council (UK), the Centre National de la Recherche Scientifique (France) or the National Institutes of Health (USA) (Austoker, 1989; Gaudillière, 2002; Picard, 1990; Soon Park, 2008; Timmermann, 2008). However, there is an ongoing debate on the potentials and limitations of these studies to capture more global transnational trends in the development of biomedicine (e.g. De Chadarevian & Strasser, 2002). The way in which emerging technologies such as cell culture, the electron microscope or, more recently, recombinant DNA have transformed and been transformed by biomedical practice at different stages of the 20th century is another main concern of the literature on biomedicine (Clarke & Fujimura, 1992; Jordan & Lynch, 1992, 1993; Landecker, 2007; Rasmussen, 1997). Scholars have also combined historical and sociological methods to explore the complex interactions between the medical and the biological in the development of treatments, diagnostic instruments and clinical trials (Keating & Cambrosio, 2007; Löwy, 1993, 1996; Pickstone, 1992).

This paper considers two interwoven stories in one locality to show the interactions between a long-term research programme and the reorganisation of biomedical sciences in a major British university. I draw on several of the research problems and investigations just discussed, and connect them in ways which may point to more integrated histories. For this purpose, I address a particularly unexplored field: the development of the research on bone, tendon, cartilage and other connective tissues aimed to attach different and distant parts of the body.

I focus on a team at the University of Manchester which started working on connective tissue after World War II. Its research was initially centred on rheumatism and arthritis, but gradually evolved to a more general programme named cell-matrix biology in the 1980s. This renaming coincided with a profound institutional reform which boosted biology in Manchester and led to the emergence of a new School of Biological Sciences at the University. The connective tissue group expanded in this new institutional setting and in 1996 formed a Wellcome Trust Centre for Cell Matrix Research. In this new Centre, the Manchester researchers utilised long-standing tools for the study of connective tissue together with newer recombinant DNA techniques.

By following the trajectories of the connective tissue researchers and their technologies, I will document the transformation of a field which emerged with a clear clinical motivation and evolved towards a more fundamental biomedical agenda. Crucially, I will analyse the interactions between institutional transformations, the introduction of new techniques, and the changing practices and concerns of the Manchester scientists. The study will be focused on a local setting—the University of Manchester—but will explore the exchanges and interactions between its researchers and other institutions in Britain and the United States. The broad chronological scope selected for this investigation (late 1940s to mid 90s) will allow me to integrate the scholarship on post-World War II with that on biotechnology and show how the genealogies of the connective tissue researchers were affected and reciprocally affected wider transformations in biomedicine.

My point of departure will be oral histories and the published papers of the connective tissue researchers. These sources will be combined with funding applications, laboratory protocols and other archival materials which will show how the Manchester researchers conducted their work and presented it to biomedical funding agencies. I will also build on the rich historical research on science and technology in Manchester (for guides see Pickstone, 1989, 2007b; Pickstone & Butler, 2005), as well as previous studies on modes of work and the transformation of research programmes. This historiography operates at various scales: from big picture history of science, technology and medicine (Pickstone, 2000, 2007a), through comparative studies of recent academic biology in Britain (Wilson & Lancelot, 2008), to a detailed analysis of the organisational politics of the University of Manchester (Wilson, 2008).^1^

The paper will be organised in three parts:

– Part I will explore the status of connective tissue research as introduced to Manchester in the late 1940s and its development up to the late 70s—for which period, Manchester biomedical research was relatively weak compared to physical sciences and clinical medicine (Pickstone, 2007b; Wilson & Lancelot, 2008).
– A major academic restructuring during the mid 1980s strengthened the biomedical departments in Manchester; it was intended to clear the way for the introduction of the new techniques of molecular biology (Wilson, 2008; Wilson & Lancelot, 2008). Part II of my paper will follow these transformations and the migrations of the connective tissue scientists to the United Sates in order to learn new recombinant DNA methods.
– Part III will focus on the return of the Manchester scientists and the combination of recombinant DNA with other techniques for the analysis of connective tissue. This resulted in the redefinition of connective tissue as *cell-matrix biology* and the creation of a centre funded by the Wellcome Trust.

## 2 Rheumatism and the origins of connective tissue research

The first incarnation of connective tissue research in Manchester was the Centre for Chronic Rheumatic Diseases, created in 1947 with a grant from the Nuffield Foundation. Rheumatism and arthritis had been main concerns in Britain and other Western countries since World War I. They were widespread in the trenches and often associated with tuberculosis, pneumonia and heart diseases. During the 1920s and 30s, rheumatism and arthritis were recognised as common diseases of industrial workers and as major causes of unemployment, prompting the foundation of disease specific charities, notably the Empire Rheumatism Council (ERC) (Cantor, 1991; Dixon, 2000, pp. 13–15; Schubert & Hamerman, 1968, pp. vii–x).

This history, and the renewed importance of rheumatism and arthritis among troops during World War II, led the British Government to place both diseases among the priorities of the National Health Service, which was created after the war to rationalise and support healthcare. Basic biomedical research was also fostered, as it was in Continental Europe and the US. The main research funders of arthritis and rheumatism in Britain were the Medical Research Council and the health-related charities. Lord Nuffield, a leading businessman from the motor industry, used his Foundation to support specialised research institutions in various British universities and hospitals (Cantor, 1991, pp. 228–231; Dixon, 2000, pp. 128–129). The Manchester Centre was one of them and among its first initiatives was a clinical trial with cortisone, a hormone which was established as a potential therapeutic agent in 1948 (Cantor, 1992, pp. 165–167).^2^

The Rheumatism Centre was located in the University’s Faculty of Medicine and associated with the Royal Infirmary, the city’s main teaching hospital. Its Chairman, Jonas Kellgren, became the first Professor of Rheumatology in Britain, thanks to an endowment from the ERC. Kellgren was a clinician who had served in World War II and was interested in the epidemiology of rheumatic patients: part of his Manchester research was on coal miners from the surrounding region of Lancashire. In his study of the organisation of biology in Manchester, Duncan Wilson has shown that the Centre was conceived as combining clinical practice and fundamental biological research. Kellgren did so by recruiting investigators from several departments of the University (Wilson, 2008, pp. 84–85).

The main research object at the Centre was collagen, a fibrous protein which was by then considered the most important component of connective tissue.^3^ Collagen had been the focus of microscopic observations since the 19th century and with the development of biochemistry, researchers attempted to determine its composition, as they were doing for other proteins during the first decades of the 20th century. Collagen was difficult to investigate because it was not soluble, but prominent protein chemists such as Albert Neuberger and Ralph Consden were able to apply the newly invented techniques of amino acid analysis and column chromatography (Piez, 1997, pp. 85–90; Grant, 2007, pp. 204–205).

Another approach to collagen in the early 20th century was X-ray crystallography, pioneered by William Henry Bragg and his son Lawrence. In the 1920s and 30s, William worked in London, whilst Lawrence had succeeded Ernest Rutherford as Professor of Physics at Manchester. Most of the Manchester work on X-ray crystallography was on inorganic compounds, and some was industry-related, but there seems to have been little connection with local industrial research, perhaps because its focus was cotton (and thus cellulose). It was across the Pennine Hills at Leeds, for wool textiles rather than cotton, that William Astbury harnessed X-ray crystallography for the study of industrially relevant fibres, among them collagen.^4^

Astbury, a former student of W.H. Bragg, attempted to find a correspondence between the sequence of amino acids and spatial conformation of collagen (Astbury, 1940, 1957, 1961; Bernal, 1963, pp. 14–15; Wilson, 2008, pp. 83–84). Between the 1930s and 50s, a number of X-ray crystallographers proposed tentative structural models for collagen, among them Linus Pauling at Caltech and J.T. Randall at the biophysics unit of King’s College London—the same department where Maurice Wilkins and Rosalind Franklin were investigating the structure of DNA (Pauling & Corey, 1951; Randall & Fitton, 1953). In 1955, Indian biophysicist G.R. Ramachandran published a triple helical model for collagen shortly before a similar one was proposed by Francis Crick and Alexander Rich (Ramachandran & Kartha, 1955; Rich & Crick, 1955; Sarma, 1998, pp. 43–62). This model resembled the double helical structure that Crick had postulated for DNA with James Watson two years before (Watson & Crick, 1953a, 1953b).^5^ Such was the proliferation of models at that time that it was agreed that crystallographers were only entitled to one proposal per molecule (Rich, 1998).

Work at the Rheumatism Centre in Manchester was inspired by these early investigations on collagen. The lines of research involved characterisation of collagen in bone and cartilage of rheumatic patients, applying tissue culture techniques and drawing on the strong tradition on histopathology at the University’s Faculty of Medicine (Baker, 1956; Wilson, 2008, pp. 8–11). Researchers also used the electron microscope, which derived from war-related work on physics and was then spreading in biomedicine. This apparatus helped bridge cytology and histology (Rasmussen, 1995, pp. 385–390, 1997, ch. 3), and to place the investigations on collagen within the emerging field of cell biology. The Manchester researchers originally used the electron microscope in cooperation with Astbury, but gradually developed their own expertise and an independent University department of Medical Biophysics—engaged with the characterisation of collagen and other biological molecules (Kellgren, Ball, Astbury, Reed, & Beighton, 1951; Pullan et al., 1983). Kellgren also established a line of research on collagen biochemistry at the Rheumatism Centre.

### 2.1 The collagen group and the biosynthesis systems

The biochemical research at Kellgren’s Centre was conducted by David Jackson, who after war service in the Air Force took a degree in Manchester jointly taught by the Departments of Chemistry and Physiology (Wilson, 2008, pp. 84–85). That both of these departments taught biochemical students reflects the historic strengths of Manchester in both chemistry and medicine, and contrasts with the situation in Cambridge, where biochemistry and other preclinical disciplines flourished and differentiated more easily. Jackson was hired by Kellgren shortly after the foundation of the Centre and in 1957 left Manchester for a long postdoctoral stay in the US. He worked with the surgeon Englebert Dunphy at the Boston City Hospital and at the University of Oregon on wound healing—another property of collagen.^6^

**Fig. 1:**
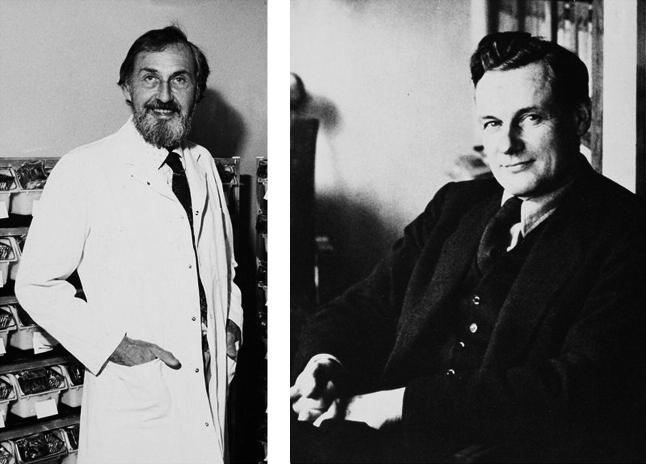
David Jackson (left) and Jonas Kellgren (right), researchers at the Centre for Chronic Rheumatic Diseases (University of Manchester Archives. Reproduced by courtesy of the University Librarian and Director, The John Rylands University Library, University of Manchester, UK).

When Jackson returned to Manchester in 1965 he organised his own research group focused on the biochemistry of connective tissue. It was located in the Department of Medical Biochemistry, which had recently been split off from Physiology. The group’s work initially involved composition studies of collagen and its surrounding molecules, using enzymes, chromatography and amino acid analysis (Grant & Jackson, 1968) (see Fig. 1).

One of Jackson’s first recruits was Michael Grant, a plant biochemist educated at the University of Manchester Institute of Science and Technology—UMIST, semi-independent from the University—7and then in Oxford (Wilson, 2008, p. 85). Grant had been invited to an interview for a position in plant biochemistry at another department of the University, but ended up accepting a post in Jackson’s group. Jackson was particularly interested in Grant’s expertise in cell culture and his techniques for the analysis of large sticky molecules of the plant cell wall. At that time, Jackson was planning to expand his research from structural to more metabolic biochemistry, including the problem of “collagen synthesis” (Grant, 2007, p. 204).^8^

Grant recalls that when he arrived in 1966 as an Assistant Lecturer, the group was small and mainly focused on teaching. Shortly after his arrival, he applied for a stay in a laboratory in the University of Pennsylvania, associated with the Philadelphia General Hospital. It was led by Darwin Prockop, then emerging as a key figure in collagen research and working on the problem of synthesis through an innovative cell culture technique (Grant & Prockop, 1972). It was with Prockop, in the grant-oriented culture of the United States, that Grant learned “how to do research”. On his return to Manchester in 1972, he too studied the synthesis and secretion of collagen.^9^

The development of cell culture techniques during the 20th century—and of antibiotics after WWII—allowed biologists to maintain cells relatively easily, so they could be used as research objects, and circulated between laboratories (Landecker, 2007, chs. 3–4). Grant’s new investigations in Manchester involved extracting cells from tendons of early chick embryos and forming collagen “synthesising systems”, first *in vivo* and then *in vitro*. He also labelled collagen amino acids with radioactive isotopes in order to detect them (Grant, 2007, p. 208; Grant, Kefalides, & Prockop, 1972, 1973). Synthesising systems had been originally conceived and used in hospital-related investigations on cancer and during the 1950s and 60s, they gradually spread to become a common tool among biochemists (Rheinberger, 1993, 1997, chs. 3–4). Radioactive isotopes had been widely introduced in biomedicine after WWII (Creager & Santesmases, 2006).

During the second half of the 1970s, Grant started to explore “the control of collagen biosynthesis” and the roles of genes in the formation of the protein. His research then shifted to the “isolation and characterisation” of collagen’s messenger RNA in order to analyse its translation to protein (Grant, 2007, pp. 207–208; Cheah & Grant, 1982; Harwood, Grant, & Jackson, 1975). Grant’s new research did not initially involve altering the structure of the molecules, though new recombinant DNA and RNA techniques were then emerging in molecular biology. It was in part, however, to facilitate the local development of molecular biology that Grant became one of the agents in a profound transformation of biomedical research at the University of Manchester.

## 3 The reorganisation of Manchester biomedicine

The reform of the University of Manchester has been studied in detail by Duncan Wilson and Gaël Lancelot. Up to the late 1970s, the major scientific strengths of the University were in chemistry and physics, with departments located at the Faculty of Science and strongly connected to the local industry. Clinical work and teaching at the Faculty of Medicine were also praised. However, the biological and preclinical sciences were relatively weak, compared to Cambridge, Oxford and University College London, as well as some new British universities where ambitious biology departments had been created.^10^

Manchester’s Zoology and Botany Departments were marginal in the Science Faculty, and found cooperation difficult. In the Medical Faculty, research in anatomy, physiology, pathology and bacteriology was often overshadowed by the clinical interests and was less of a priority than service work for medicine. These departmental structures were common in provincial European and British universities, many of which undertook parallel reforms (Lancelot, 2007; Wilson & Lancelot, 2008, pp. 105–107).

The split between the Medical and the Science Faculty hindered the formation of biomedical programmes in Manchester. This was especially true in biochemistry, for which two departments had emerged at the University: Medical Biochemistry from Physiology, and Biological Chemistry from a large Chemistry department with significant historic strengths in organic analysis and synthesis, as well as in fermentation studies—Chain Weizmann developed there the fermentation process to produce acetone, a substance of paramount importance for military operations during World War I.^11^ Staff in Medical Biochemistry and Biological Chemistry had limited interactions: they lived in different buildings, were administered by different Faculties, and used different research facilities (Wilson, 2008, pp. 14–15).

The Departments of Botany and Zoology had attempted to collaborate over genetics and cell biology, but personal animosities interfered. For the most part, staff in the two departments pursued structural analyses and comparative physiological research on plants and on animals, rather than the studies in cytology, cell physiology, genetics or immunology which were elsewhere bringing biology departments closer to preclinical studies. Though some zoologists and botanists shared an electron microscope with Jackson’s group, there was little collaboration (ibid., p.15).

Manchester biomedicine was radically reconfigured in the 1980s for three main reasons. Firstly, Botany, Physiology, Medical Biochemistry and a new department of basic Dental Sciences came to be led by young professors—among them Grant—who agreed on a reform agenda. Secondly, a microbiologist, Mark Richmond, was appointed Vice-Chancellor of the University; as a former Government Advisor on genetic manipulation, he considered that the biomedical sciences could only prosper by using the new recombinant DNA techniques that had emerged in molecular biology. Thirdly, thanks to increasing budget cuts imposed by the new Government of Margaret Thatcher, the research outputs of university departments came under increasing scrutiny. National mechanisms were created to compare outputs and then to fund accordingly. That Manchester’s biological and medical sciences ranged from average to poor undercut the arguments of those who did not favour reorganisation (ibid., ch. 3; Wilson & Lancelot, 2008, pp. 97–101).

Following Jackson’s retirement in 1981, Grant became head of Medical Biochemistry and of the connective tissue group. By that time, the Department of Biological Chemistry had been renamed Biochemistry. In 1982, along with Christopher Pogson, the head of Biochemistry, Grant arranged to create a unified department that was jointly administered by the Faculties of Science and Medicine, and named Biochemistry. Four years later, in 1986, the young professors and Richmond agreed a major reorganisation of biomedical sciences. A School of Biological Sciences was created with four departments: (1) Biochemistry and Molecular Biology; (2) Cell and Structural Biology; (3) Physiological Sciences, and (4) Environmental Biology.

The previous departments, in both the Faculties of Science and Medicine, were dissolved and their staff asked to redistribute themselves among the departments of the new School. Almost all the researchers in the unified Department of Biochemistry joined Biochemistry and Molecular Biology, whereas the staff from the Faculty of Medicine mainly went to Physiological Sciences. Most of the botanists and zoologists went respectively to Cell and Structural Biology and to Environmental Biology (Wilson, 2008, pp. 24 and ff.; Wilson & Lancelot, 2008, pp. 98 and ff.).

The reform was well received within the University except for the Department of Anatomy, whose staff did not wish to subordinate their identity to molecular biology. John Scott, a researcher who had joined Jackson’s group in the mid 1970s, with a new Chair of Chemical Morphology, moved to the Chemistry Building in 1986, outside the new School of Biological Sciences. He maintained a biochemical and biophysical approach to connective tissue using electron microscopy, but not incorporating recombinant DNA techniques (Scott, 2007). Staff in the new department of Environmental Biology—which hosted most of the zoologists—felt unappreciated, partly for not introducing recombinant DNA methods; most of its researchers left the University after a second reform in 1993, which dissolved the departments of the new School and created a Faculty of Life Sciences without departments, just research groups (Wilson & Lancelot, 2008, pp. 101–105).

It is worth noting that neither Richmond, nor any of the young professors were themselves molecular biologists. They were a new generation of researchers aiming to apply the new recombinant DNA techniques to problems they were already investigating from other perspectives. They did not seek to create a molecular biology centre or department, like the Laboratory of Molecular Biology of Cambridge (LMB) or the Department of Molecular Biology of the University of Edinburgh, founded during the 1960s (De Chadarevian, 2002). Instead, Manchester and other British and European universities—such as Sheffield or Paris—created unified biomedical schools or departments where recombinant DNA and other techniques of molecular biology were introduced as shared resources and oriented towards the research interests of each group (Lancelot, 2007; Wilson & Lancelot, 2008, pp. 105–107).^12^

By looking at Manchester, or the other universities which adopted similar reorganisations, one can see that the reform helped redefine programmes which were initially outside the scope of molecular biology and the new recombinant DNA techniques. One of these programmes was connective tissue research, which started adopting recombinant techniques in the late 1980s. By studying the genealogy of these techniques and their introduction into Manchester, one can see how the dialogue between recombinant DNA and pre-existing problems in the field of connective tissue shaped patterns of work within and beyond the new organisation of the University.

### 3.1 The transformation of collagen research

The University reform coincided with major changes in the work of the connective tissue group. From the later 1970s, Grant and his co-workers increased their focus on messenger RNA (mRNA) in their *in vitro* synthesis systems. They aimed to isolate the mRNA involved in the formation of different types of collagen (Cheah & Grant, 1982; Harwood et al., 1975).^13^ Within this line of research, Grant hired as a postdoctoral fellow Raymond Boot-Handford, who had completed a PhD on the metabolism of diabetes at University College London. They started working on the role of the collagen which coats the blood vessel walls in heart complications following diabetes. Thus the move towards nucleic acids coincided with a broadening of the clinical interests of the group, from rheumatism to diabetes and other diseases which were becoming priorities in the 70s and 80s, within the struggle against obesity and other emerging health problems in Western societies.

Their interest in nucleic acids remained, however, subordinated to protein chemistry. During the first half of the 1980s, mRNA was used to access the different collagens and their alterations in diabetes and other diseases. One of the first experiments of Grant and Boot-Handford was to measure rates of collagen synthesis in systems obtained from diabetic and healthy rats. Grant also began to use monoclonal antibodies to react with the collagen alterations caused by diabetes (Grant, 2007, p. 209).

Both investigators became increasingly persuaded that to develop their programme it was necessary to shift the focus of research “from the perspective of proteins to that of genes”. The first proposals of a University reform and the emphasis of such proposals on recombinant DNA led them to believe that if the genes responsible for diabetes could be isolated, then finding their mutations would be simpler than looking for the resulting proteins. DNA and RNA could by then be tackled in the laboratory thanks to the emergence, among others, of complementary (cDNA) cloning and sequencing techniques which allowed researchers to obtain DNA fragments and to analyse their nucleotide structure. These techniques, however, were only vaguely known by the Manchester researchers.^14^

Grant decided that Boot-Handford should apply for a stay in the laboratory of Darwin Prockop, the researcher from whom he had himself learned the collagen synthesis techniques. Prockop’s group had significantly changed since Grant’s stay in the early 1970s. In 1973, it had moved to the Rutgers Medical School in New Jersey, where Prockop had been appointed Director of the Centre for Human Molecular Genetics. The group in Rutgers used recombinant techniques extensively for obtaining and characterising the DNA fragments which constituted the genes producing defective collagens in various diseases. Boot-Handford describes the laboratory after his arrival, in 1985, as a large-scale organisation with “visitors from all around the world” learning the techniques.^15^

The introduction of recombinant DNA into Prockop’s group presented complex genealogies. Up to the late 1970s, the team had continued to work on collagen synthesis and some of its members were unenthusiastic about the emergent recombinant techniques. Bjorn Olsen, one of Prockop’s first collaborators, recalls that shortly after moving to Rutgers he was asked to teach *Nucleic Acid Biochemistry* to medical undergraduates, a course which other members of the group regarded as “boring” and unrelated to collagen research. This indifference, according to Olsen, was due to the fact that by the mid-late 70s the dominant perception among connective tissue researchers was that the recombinant DNA techniques only worked in microorganisms. At that time, “it was not clear” to them whether these techniques would be suitable to chicks, rats, or the other complex multicellular organisms with which connective tissue scientists normally worked.^16^

This perception reflects the problematics in the circulation of techniques between two largely non-interacting communities: molecular biologists and connective tissue researchers. Despite Francis Crick having proposed a triple-helical model for the structure of collagen in the mid 1950s (see above), he subsequently identified his research trajectory and the disciplinary formation of molecular biology with DNA, its double helix and the further achievements around this molecule. In his autobiography, he states that despite the apparent similarities between both helical structures, “in a very real sense collagen is not as important a molecule as DNA” (Crick, 1988, p. 67). Crick and other self-declared founders of molecular biology—including Rich—have retrospectively considered collagen only an incidental interest in their careers.

Connective tissue researchers, reciprocally, remained only distantly aware of the developments in molecular biology and this led them to associate recombinant DNA with the microorganisms—bacteria or bacteriophage viruses—with which molecular biologists had worked during the golden era of this discipline in the 1960s. However, by the time Olsen was preparing his undergraduate syllabus,^17^ molecular biologists were gradually shifting their research interests to multicellular organisms. This move, known as the “the mass migration”, shaped the first attempts to producing recombinant molecules, which combined microbial DNA with fragments obtained from frogs and other complex eukaryotic organisms (Morange, 1997; Yi, 2008, pp. 383 and ff.).

During the mid 1970s, molecular biologists were also increasingly urged by political authorities to find medical applications to their techniques. The US President Richard Nixon had declared the war on cancer and, by the end of the decade, the first biotechnology companies were founded, with the explicit aim of finding diagnostic and pharmacological applications of recombinant DNA. This triggered a large restructuring of biomedical complexes in the US and Europe, and led researchers from different disciplines to adopt the recombinant techniques (Gottweis, 1998b, pp. 107 and ff.; Kenney, 1986; Wright, 1998, pp. 94–95; Yi, 2008, pp. 592–593).

Human geneticists were among the first in introducing recombinant tools. The new medical and eukaryotic approach of molecular biology led them to initiate a “hunt” of the genes causing different hereditary diseases. Such gene location had been a permanent goal in the history of genetics and researchers had developed different non-molecular means throughout the 20th century: statistics, family pedigrees and chromosomal images, among others (Kevles, 1995 [1985], pp. 264 and ff., 1992; Cook-Deegan, 1994, ch. 2; Harper, 2008). Human genetics also seems to have provided the bridge through which recombinant DNA permeated connective tissue research.

At the same time Olsen was learning and teaching molecular biology (1975 and 1976), Prockop left Rutgers for a temporary stay at the National Heart and Lung Institute of the National Institutes of Health (NIH), a federal structure of government laboratories with substantial expertise in microbiology and human genetics. The seminar series at each of the specialist Institutes were open to all researchers and Prockop, who had initially visited to study the role of collagen in heart and lung diseases, recalls having learned key techniques from another group working on atypical haemoglobins.^18^ This group sought to isolate and analyse the gene causing sickle cell anaemia. For this purpose, its researchers applied to mRNA of patients complementary (cDNA) cloning and determined the nucleotide sequence of the resulting DNA fragments.

Prockop was enthusiastic about applying recombinant techniques to collagen when he returned to Rutgers. In 1980 and 1981, he wrote a number of articles advocating cloning and sequencing as the future for connective tissue disease research (Prockop, 1980). The techniques for the study of proteins, such as amino acid analysis, could become “obsolete” in the face of the new approach (Prockop, 1981). This feeling was common in other areas of biomedical research—such as immunology or developmental biology—which began focusing on genes isolated through recombinant DNA rather than on their protein products (García-Sancho, 2008, pp. 162–166; Gilbert, 1996; Löwy, 1996; Morange, 1997, pp. 389 and ff.; Tauber & Podolsky, 2000).

In 1980, Prockop organised an international meeting on “gene families of collagen” which brought to Rutgers haemoglobin scientists, experts in recombinant DNA and collagen researchers, some of whom had already started to clone and sequence genes for this protein (Boedtker, Crkvenjakov, Last, & Doty, 1974; Lehrach et al., 1978; Prockop & Champe, 1980). Prockop himself hired Francesco Ramirez, an expert in cDNA cloning who had been working at Columbia University on the application of this technique to haemoglobin. The Prockop group then focused on cloning and sequencing the genes responsible for collagen diseases such as the bone condition *Osteogenesis imperfecta*. Olsen, who at that time headed a neighbouring laboratory at Rutgers, also abandoned his previous work on electron microscopy and took up the cloning and sequencing of collagen genes.^19^

Prockop did not interact directly with molecular biologists until, in 1986, he moved back to Philadelphia to chair the Department of Biochemistry and Molecular Biology at Jefferson University. Boot-Handford had arrived a year before; in both New Jersey and Philadelphia he learned how to measure levels of mRNA expression—his initial goal—and how to make and screen cDNA libraries. Upon his return to Manchester in the late 80s, he abandoned rats and diabetes, and associated again with Grant to obtain cDNA clones of the gene for a specific type of collagen—collagen X—which was involved in bone formation. They were now competing with Olsen’s group at Rutgers. In 1991, the Manchester team was the first to isolate the bovine gene and then the human (Thomas, Kwan, Grant, & Boot-Handford, 1991; Thomas, Cresswell, et al., 1991b).

When asked why he did not move to a British molecular biology institution to learn the recombinant techniques, Boot-Handford argues that Prockop’s group was already known by Grant and “spoke the same language”.^20^ This highlights the complexity of the trajectories in the introduction of recombinant DNA, the role of professional networks and the importance of “centres of calculation” (Latour, 1987, ch. 6). Furthermore, in their circulation, the new techniques interacted with pre-existing lines of research and were applied to problems of connective tissue, such as collagen synthesis.

The adoption of cloning and sequencing by connective tissue researchers was, thus, negotiated in complex ways during the mid and late 1970s. The new techniques were repackaged in a discipline—human genetics—whose medical orientation and model organisms—rats and humans—were closer to the interests of connective tissue researchers. Molecular biologists, additionally, had adopted multicellular organisms and medical concerns, and this widened their research scope, displacing their “descriptive level from the molecule to the cell” (Morange, 1997, p. 369). This displacement and subsequent redefinition of the cell was crucial in the new identity that connective tissue research adopted in the 1980s.

## 4 From connective tissue to matrix biology: the redefinition of a research field

Boot-Handford returned to Manchester in 1987, shortly after the reorganisation of the biomedical sciences in the University. Following the creation of the new School of Biological Sciences (1986), the connective tissue team was assigned to the Department of Biochemistry and Molecular Biology and renamed Extracellular Matrix and Tumour Biology Group. The term *matrix*, despite having a long history, had significantly changed its meaning in the 80s and developed hand-to-hand with the transformations of connective tissue research.

The first uses of *matrix* date from the 1930s to designate the conglomerate of proteins, sugars and other components which make up connective tissue (Piez, 1997, pp. 87–88). The term spread among researchers during the 50s and 60s, and was present in textbooks on connective tissue and its diseases. One written in 1968 referred to *matrix* as a “formless jelly-like” substance which surrounded the cell and helped it anchor onto other cells in order to form cartilage, tendons or bones (Schubert & Hamerman, 1968, p. 1). A decade later, adjectives such as formless, inanimate or inert were abandoned, as the matrix came to involve a rich variety of molecules, and to be characterised by a “dynamic interaction” with the cells (Piez, 1997, pp. 90–91). Matrix biology was then defined as the field studying those interactions (Hay, 1982, p. 1).

Researchers involved in matrix biology during the 1980s and 90s unanimously point towards the key role of recombinant DNA techniques in revealing the complexity of interactions between cell and matrix, as well as between the diversity of components of connective tissue. Nevertheless, they also stress the importance of the “multicellular perspective” of the field in order to analyse the two-directional connections not only between cell and matrix, but also from cell to cell through the matrix.^21^

If then, matrix biology was considerably helped by the recombinant techniques to acquire its new identity, this identity remained crucially different from that molecular biology was acquiring between the 1970s and 80s. Whereas at that time, molecular biologists were mainly concerned with the interactions between the cell nucleus and the cytoplasm, matrix biologists considered two-directional connections between cells, using the extracellular space. The emerging recombinant techniques were being presented by some molecular biologists as tools unifying biology (e.g. Hood, 1990), but the sharing of methods could be understood in different ways. Matrix research was not seen by its practitioners as a province of molecular biology, but as an independent field with its own evolving problems.

The Manchester Extracellular Matrix Group was an institutional materialisation of this differential identity. In the mid-late 1980s, shortly after the renaming of the group, Grant persuaded Boot-Handford and other former Manchester connective tissue scientists to apply for funding to return to the University (Wilson, 2008, pp. 85 and ff.). They chose the Wellcome Trust as the most suitable funding body, though the group’s prior fellowships and grants had been mainly from the Medical Research Council (MRC) and the Arthritis and Rheumatism Research Campaign, the new name of the Empire Rheumatism Council. New private or charitable foundations, with different health priorities, were rivalling the post-World War II charities and the MRC as the main funders of British biomedicine.^22^

The other two researchers whom Grant decided to sponsor reflected the wide array of instruments, practices and experimental approaches gathered in matrix biology. Martin Humphries had migrated to the National Cancer Institute of the NIH in the early 1980s following a PhD at Manchester University’s Department of Biochemistry, which he started before the merger with Medical Biochemistry and integration with Grant’s group. He had worked on the protein fibronectin, also part of connective tissue and involved in cell growth and adhesion. Karl Kadler, like Boot-Handford, had worked at Prockop’s laboratory, but his focus was on electron microscopy of collagen. His PhD had been at the University’s Department of Medical Biophysics, which like Grant and Jackson’s group had been founded by former researchers of the Rheumatism Centre. Neither Humphries nor Kadler used recombinant DNA regularly: for them it was “a complement” rather than the main tool of their research.^23^

In their applications for Wellcome funding, Humphries and Kadler both mentioned recombinant techniques, but as ancillary to other tools to be incorporated to the Extracellular Matrix Group. Humphries sought a “combination of peptide chemistry, cell biology and molecular biology approaches” to study a region of fibronectin linking the protein to the cell. In the NIH, he had used cDNA cloning to obtain the gene for a particular fibronectin, but his main approaches in the study of the protein were biochemistry and cell biology.^24^ Kadler worked with enzymatic cleavage and mutant collagen, occasionally using sequencing. But in his Wellcome application he stressed biophysical approaches to the structure of collagen fibres, using light and electron microscopy to study the assembly of the protein.^25^

The Wellcome approved both applications, and Kadler and Humphries joined Boot-Handford as returnees to Manchester between 1987 and 89. In the early 1990s, Grant proposed to the Wellcome the establishment of a reference centre for cell matrix research in Manchester. At that time, the Wellcome was beginning to sponsor national specialised centres in different fields, such as developmental biology in Cambridge and human genetics in Oxford.^26^ The Manchester bid was issued in 1993 by Grant, Kadler, Humphries and John Sheehan, another researcher in the matrix group.

The centre bid stressed the “strengths” of the Manchester group in the “chemistry, biophysics, cell biology and molecular biology” of the extracellular matrix. The Manchester researchers, according to the application, possessed expertise in the technologies of “electron microscopy, structural molecular biology and image analysis, protein crystallography, recombinant DNA technology, transgenic animal techniques, molecular graphics and computation, and high field n.m.r. [nuclear magnetic resonance]”.^27^ Structural molecular biology and recombinant DNA were two among many disciplines and tools. They may be seen as key exemplars of the profusion of analytical ways of knowing and working which were now characteristic of biomedical research and used not only in molecular biology, but across many other fields (Pickstone, 2007a, especially pp. 513–514).

**Fig. 2:**
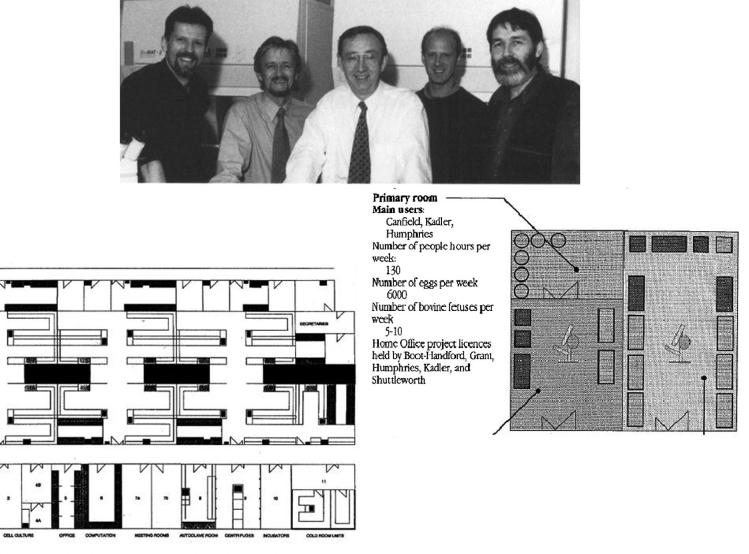
Plans for the new Wellcome Centre for Cell Matrix Research at the University of Manchester (bottom left) and one of the proposed shared laboratories, as conceived in 1993 (bottom right). Top, from left to right, Martin Humphries, Tim Hardingham, Michael Grant, Karl Kadler and John Sheeham, some of the cell matrix researchers (Michael Grant’s personal archive, Faculty of Life Sciences, University of Manchester, UK, and Wilson, 2008, p. 87. Reproduced with permission).

The group proposed a series of shared laboratories at the Matrix Centre devoted to “biomolecule assembly and dynamics”, “cell culture” and “biomolecule purification and analysis”. The former facility was devoted to electron microscopy and biophysics approaches, while the latter included a DNA synthesiser, and other protein sequencing and analysis techniques. The cell culture suit was aimed to produce mRNA and protein molecules to be analysed (see Fig. 2).^28^ Here we also see synthetic molecular biology techniques combined with analytic instruments from biophysics, biochemistry and cell biology, and all focused on the study of the extracellular matrix. The new automatic DNA synthesisers were being incorporated to the historical strengths of the Manchester researchers in cell culture and electron microscopy, and this resulted in the redefinition of connective tissue as both an object and a research field.

Pat Goodwin, one of the Wellcome Trust officers involved in processing the application, has claimed that the main reason for its success was the “fine combination” of recombinant DNA with instruments and perspectives “from biophysics”. The rise of the recombinant techniques in the preceding decades had led most contemporary applications to use them in reductionist studies, mainly focused on the analysis of DNA. The Manchester bid, on the contrary, presented such techniques as tools to be combined with others in an integrated approach to the cell matrix. This argument is reinforced by Ramirez, Prockop’s former collaborator, who reviewed the application and considered it the beginning of a specific line of research pursued by Manchester without the necessity of “following others”.^29^

The Wellcome Trust Centre for Cell Matrix Research opened in 1996. Its foundation represents the emergence of a biomedical field derived from connective tissue research but shaped by a new configuration of tools, institutions, patronage and research orientation. This particular configuration, and its opportunity within the transformation of British biomedicine, was essential for the success of the Matrix Centre over other institutions^30^ and the rise of the biomedical sciences in Manchester.

### 4.1 Automation and spread of recombinant techniques

The new Centre for Cell Matrix Research, as the School of Biological Sciences had done the decade before, emphasised the use of recombinant DNA in its publicity when it opened (Wilson, 2008, pp. 86–89). However, these techniques remained initially confined to Boot-Handford and Grant’s team within the Manchester matrix biologists. Grant and Boot-Handford incorporated human geneticist Gillian Wallis and biochemist Cay Kielty to their group before the move to the new Centre. During the early 1990s, they used cloning and sequencing to construct cDNA libraries of the chick and human gene for collagen type X (Thomas, Kwan et al., 1991; Thomas, Cresswell, et al., 1991). The sequence and expression patterns of the gene were then analysed by Boot-Handford (Kong et al., 1993; Thomas et al., 1995).

The expertise in sequencing and cloning gave Boot-Handford considerable influence, as “the only British scientist in the field” who had mastered the techniques. At that time, sequencing and cloning were labour-intensive procedures described by their inventors in protocols and textbooks. They could be taught at the bench and circulated from one researcher to other. During the first half of the 1990s, Boot-Handford taught sequencing and cloning to other University staff, especially in the areas of pathology and clinical medicine. Nevertheless, most of the Extracellular Matrix Group was initially uninterested in his teaching: the older researchers resisted the potentialities of recombinant DNA, while most of the young staff “did not need the techniques” for their work.^31^

The team created by Humphries soon became another focus of techniques from molecular biology. However, the key technical expertise was derived from London—and again from genetics—rather than from Humphries’ stay at the NIH. In the mid 1990s, Humphries hired a former undergraduate colleague, Linda Green, with the specific aim of applying the Polymerase Chain Reaction (PCR) to the fibronectin project.^32^ Green had learnt this method at the Middlesex Medical School, in the context of genetic diagnosis of brain tumours. Her job was isolating mRNA in rats and multiplying it with PCR, in order to form cDNA libraries and detect the markers of the tumours through Southern Blot—i.e. complementary DNA probes.

**Fig. 3:**
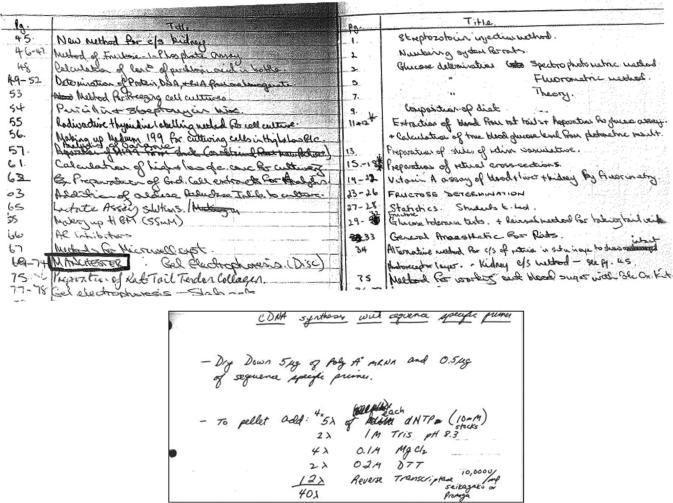
List of protocols used by Boot-Handford during the 1990s in Manchester (above) and one of his cDNA synthesis procedures (below) (Raymond Boot Handford’s personal archive, Wellcome Trust Centre for Cell Matrix Research, University of Manchester, UK. Reproduced with permission).

Green’s work at the matrix group, like Boot-Handford’s, relied on manual protocols compiled during her previous job. The protocols derived from oral instructions, published papers and unpublished notes written by other researchers (see Fig. 3).^33^ They were the foundations of Green and Boot-Handford’s teaching of the techniques to other investigators. Green also devoted a substantial part of her PhD thesis—conducted after her arrival to Manchester—to the optimisation of manual PCR for the study of genes producing fibronectin (Green, 1994, pp. 73–77 and 86–91).

But as Green and Boot-Handford were teaching their protocols, automatic instruments became available for recombinant DNA work. From the mid 1980s onwards, Applied Biosystems, Cetus Corporation and other biotechnology companies marketed devices which conducted sequencing and PCR with minimum human intervention (Chow-White & García-Sancho, in press; García-Sancho, 2008, pp. 167–193; Rabinow, 1996). These instruments spread among the biomedical community and by the early and mid 90s were permeating many day-to-day laboratory activities (Jordan & Lynch, 1992; Jordan & Lynch, 1993; Keating, Limoges, & Cambrosio, 1999; Ramillon, 2007).

The Manchester Matrix Centre incorporated some of these automatic devices. The Wellcome Trust application to create the Centre included a DNA synthesiser and an automatic protein sequencer.^34^ Shortly after its submission, in 1995, Professor of Dental Genetics, Michael Dixon, led a new Wellcome grant proposal to acquire an automatic DNA sequencer for the whole Faculty of Life Sciences. In line with the philosophy of the University’s reform, the sequencer and associated equipment would be used as centralised and shared facilities.^35^ The applicants, among them Humphries, secured funds to buy a 377 DNA sequencer, which was marketed by Applied Biosystems.

The sequencer and other contemporary automatic devices led to the spread of recombinant DNA across the groups of the Matrix Centre. In 1999, Dixon issued an application for renewal and maintenance funds to the Wellcome and, by that time, five matrix teams had used or were planning to use the sequencer.^36^ The availability and increasing user-friendliness of these automatic devices made researchers less dependent on Green and Boot-Handford, and able to perform sequencing and PCR autonomously. These researchers were, at the same time, increasingly unaware of the biological mechanisms underlying sequencing and PCR, which they saw simply as tools. Thus the techniques became both more pervasive and more “blackboxed” (Latour, 1987, ch. 3). By the late 90s, Green and Boot-Handford were only called on by other matrix researchers when the devices would not work properly.^37^

## 5 Conclusions

The transformation of connective tissue research into cell matrix biology in Manchester between the 1940s and 90s constitutes a suitable case for integrating the different levels, perspectives and timeframes of STS literature on biomedicine. It shows how global shifts in the organisation of the British and US biomedical complexes—triggered by socio-political changes and the emergence of biotechnology in the late 70s—affected the local biomedical platforms integrated by Michael Grant and Darwin Prockop’s collagen research groups. The advent of recombinant DNA methods fostered institutional reforms—with new undergraduate courses, schools and university departments—and changed the configuration of tools and patterns of work of biomedical investigators. It also transformed the way in which the Manchester researchers integrated the biological and the medical.

Connective tissue research in Manchester emerged as a consequence of the rise of biomedicine after World War II and, more concretely, the medical concern with rheumatism and arthritis, including new treatments. Clinicians and MDs such as Prockop, Jonas Kellgren and Bjorn Olsen played a leading role in its development, in cooperation with biochemists, biophysicists, histopathologists and cell biologists. During the last third of the 20th century, the field gradually evolved towards more fundamental research and this was eased by the University reform, the rise of preclinical and biological sciences in Manchester, the emergence of the Extracellular Matrix Group, and the new focus on diseases such as cancer or diabetes. In this way, connective tissue research differs from the history of other post-World War II lines of biomedical research, which evolved from fundamental to the clinical (e.g. De Chadarevian, 2002).

The advent of recombinant DNA and its perception as “the right tool for the job” by funders and governments (Clarke & Fujimura, 1992) was decisive for the transformation of connective tissue research and the emphasis that investigators placed on these new technologies—partly to attract funding and prestige—may suggest that they replaced previous tools and concerns. This was not the case in the Manchester group, however, where cDNA cloning, sequencing and PCR interacted with long-standing instruments and problems in the study of connective tissue, such as cell culture, electron microscopy and collagen biosynthesis. The new techniques, moreover, had needed to be repackaged in human genetics, a medically-oriented field which was closer to the interests and model organisms of the connective tissue researchers. And they did not permeate all the lines of research on matrix biology until blackboxed in automatic devices established as centralised and shared facilities.

The Manchester case, in sum, suggests a long-term strategy of “molecularisation” (De Chadarevian & Kamminga, 1998) in which an array of instruments and biological fields interacted with a long-standing medical problem: the role of connective tissue in health and disease. It also illustrates the considerable and sometimes contested “cumulations” of analytical techniques—such as electron microscopy and sequencing—in recent biomedical research (Pickstone, 2007a, p. 515). Though these techniques could help create new technical specialisations, the sharing of methods and the common reference to genes and proteins could be claimed as a unification of biology.

We have seen that in Manchester, the new School of Biological Sciences which replaced a number of biological and pre-clinical departments was meant to focus on molecular biology, with only weak internal divisions. When we look more closely, however, we see the continuity of a research programme, acquiring new methods alongside their old methods, and retaining a clinical dimension, even as the field of study deepened. We see apparently simple structural and functional relations in cartilage re-understood as complex interactions, so that matrix biology could emerge as one key field of biology, in parallel and in connection with many other such fields. When the School of Biological Sciences was transformed into a Faculty in 1993, it was to contain research groups instead of departments. Its structure seemed to reflect the shared methods and facilities, but also the different foci and problems that gave meaning to most research careers, and which were related in complex ways to patterns of training and to disciplinary organisations such as professional societies and their journals.

For all the good work on molecular biology, historians have hardly begun to analyse these dynamic dialogues between divergent and unifying concerns, analytical and synthetic approaches, research projects and disciplines, or between patterns of work and academic (and related) structures. I have tried to show some of the interactions for one University and one research field. We will need many more cases and comparative analyses to understand the typical moves, the contextual differences, and indeed the continuities across the so-called bioscience revolution of the late 20th century.

## Footnotes

1 Other local studies which have inspired my work are those on the development of biomedicine in Spain and Italy during the second half of the 20th century (e.g. Capocci & Corbellini, 2002; Santesmases & Muñoz, 1997).

2 D. Jackson (undated) Unpublished memoirs (Michael Grant’s personal archive, Faculty of Life Sciences, University of Manchester, UK).

3 Collagen was not the only focus of connective tissue research in Manchester during the late 1940s and 50s. The University also hosted research on mucopolysaccharides, proteins surrounded by sugars which also integrate connective tissue. This latter work was conducted in both the Rheumatism Centre and other departments of the University whose connections with Kellgren’s group were sometimes weak (Scott, 2007; D. Ashhurst, interview with author, Wellcome Trust Library, London, UK, 2008).

4 Collagen was the component of commercial products which were industrially relevant during the late 19th and early 20th centuries. Apart from wool, other industries which conducted biochemical and biophysical research on collagen—sometimes in cooperation with academia—were devoted to glue, leather, meat and photographic gelatine (Ashhurst & Bailey, 2005).

5 The absence of an extended time gap between the proposals by Ramachandran and Crick–Rich gave rise to a controversy over the priority of the triple helical model of collagen. A number of commentators have argued that it is normally attributed to Crick and Rich due to their first-world institutional affiliation and popularity, given their involvement in molecular biology and the postulation of the double helix of DNA (Balaram, 2004; Sarma, 1998, pp. 63–70).

6 D. Jackson (undated) Unpublished memoirs (Michael Grant’s personal archive, Faculty of Life Sciences, University of Manchester, UK).

7 UMIST derived from the Manchester College of Science and Technology, which was owned by the City Council and linked with the University of Manchester from 1905. After World War II, the College became independent of the city government, and in 1955 adopted the name UMIST. In 2004, UMIST and the University of Manchester were merged (Wilson, 2008, pp. 15–16 and ch. 9).

8 Jackson had been interested in the synthesis of collagen since, at least, the late 1950s, but at that time referred to it as “the formation” or “the biogenesis” of collagen, quoting 19th century microscopists such as Theodor Schwann and Rudolf Virchow (Jackson, 1957 and id.: “The biogenesis of collagen”. Proof for the Conference of the British Gelatine and Glue Research, University of Cambridge, 1957. Francis Crick’s Papers, Wellcome Trust Archives, London, UK file number PP/CRI/E/1/5/3).

9 M. Grant, interview with author, Faculty of Life Sciences, University of Manchester, UK, 2008.

10 For a broader perspective of the University of Manchester—born as Owens College in 1851—see Pullan, Ford, & Leahy (2007), Pullan & Abendstern (2000, 2004), and Pickstone (2007b).

11 Research on fermentation was also a main strength of UMIST, where Grant had studied biochemistry as an undergraduate. UMIST possessed a strong tradition in biochemistry and this descended from the department of brewing science that its antecedent, the Manchester College of Science and Technology, had opened during the interwar years (Wilson, 2008, pp. 15–16; see note 7).

12 US universities such as Berkeley or Stanford also reorganised their departmental structures in the face of the developments in molecular biology. These reorganisations, however, occurred in the 1960s and 70s, preceding those in Europe (Trow, 1984; Vettel, 2004).

13 There are more than 25 known types of collagen involved in the formation of different tissues—e.g. bone, skin, cartilage or tendon. The diversity of this molecule began to be known in the mid 20th century and, from the 1970s onwards, researchers specialised in specific types of collagen (Grant, 2007, pp. 204–207).

14 M. Grant and R. Boot-Handford, interviews with author, Faculty of Life Science and Wellcome Trust Centre for Cell Matrix Research, University of Manchester, UK, 2008.

15 R. Boot-Handford, interview with author, Wellcome Trust Centre for Cell Matrix Research, University of Manchester, UK, 2008.

16 B. Olsen, interview with author, Harvard Medical School, US, 2008.

17 Olsen subsequently renamed his course *Nucleic Acid Biochemistry and Molecular Biology*. He was elected twice lecturer of the year by his students (B. Olsen, interview with author, Harvard Medical School, US, 2008).

18 D. Prockop, interview with author, Philadelphia, US, 2008.

19 Both Prockop and Olsen held a medical degree (MD). This, according to Olsen, furnished them with a “more global view” of the biology of the organism and led them to be more receptive than collagen biochemists towards the recombinant DNA techniques (B. Olsen, interview with author, Harvard Medical School, US, 2008). Molecular biologists had developed complex interactions with clinical researchers since the foundational period of this discipline in the 1950s. Soraya de Chadarevian has shown this for the investigations on the three-dimensional structure of haemoglobin and physicians working on sickle-cell anaemia (de Chadarevian, 1998).

20 R. Boot-Handford, interview with author, Wellcome Trust Centre for Cell Matrix Research, University of Manchester, UK, 2008.

21 M. Grant, R. Boot-Handford and M. Humphries, interviews with author, Faculty of Life Sciences and Wellcome Trust Centre for Cell Matrix Research, University of Manchester, UK, 2008.

22 During the late 1980s and early 90s, the Wellcome Trust was becoming a wealthier institution due to the gradual sale of the pharmaceutical company Wellcome Foundation. In 1995, the majority of the shares of Wellcome Foundation were transferred to Glaxo Laboratories for four billion dollars (Wilson, 2008, p. 83). Boot-Handford was the only early Manchester matrix researcher whose return was not funded by the Wellcome Trust, but by a fellowship from the Royal National Institute of the Blind to investigate diabetes. The application seems to have disappeared; neither the applicant nor the institutional archives involved in the process hold a copy of this material.

23 M. Humphries and K. Kadler, interviews with author, Wellcome Trust Centre for Cell Matrix Research, University of Manchester, UK, 2008.

24 M. Humphries (1987) “Characterisation of cell adhesion sites within fibronectin” (Martin Humphries’s personal archive, Wellcome Trust Centre for Cell Matrix Research, University of Manchester, UK, p. 2).

25 K. Kadler (1988) “Biochemistry of matrix in normal and diseased connective tissue” (Karl Kadler’s personal archive, Wellcome Trust Centre for Cell Matrix Research, University of Manchester, UK, pp. 3 and ff.).

26 P. Goodwin, interview with author, Wellcome Trust, London, UK, 2008.

27 M. Grant, M. Humphries, K. Kadler, and J. Sheehan (1993) “The establishment of a Wellcome Cell-Matrix Research Unit—Bid for refurbishment costs, equipment and infrastructure” (Michael Grant’s personal archive, Faculty of Life Sciences, University of Manchester, UK, p. 4).

28 M. Grant, M. Humphries, K. Kadler, and J. Sheehan (1993) “Equipment justification” (Michael Grant’s personal archive, Faculty of Life Sciences, University of Manchester, UK) and M. Grant, M. Humphries, K. Kadler, and J. Sheehan (1993) “The establishment of a Wellcome Cell-Matrix Research Unit—Bid for refurbishment costs, equipment and infrastructure” (Michael Grant’s personal archive, Faculty of Life Sciences, University of Manchester, UK, pp. 6 and ff.).

29 P. Goodwin and F. Ramirez, interviews with author, Wellcome Trust, London, UK, and Mount Sinai Medical Centre, New York, US, 2008.

30 Other British institutions working on connective tissue took different research directions between the 1980s and 90s. The Kennedy Institute for Rheumatology in London began at that time to experiment with animal models of collagen diseases, whereas the Strangeways Research Laboratory—the first home institution of Crick after his arrival to Cambridge—abandoned connective tissue to focus on genetic epidemiology. The Unit for Collagen Research at the University of Bristol maintained a veterinary orientation and its links to the meat industry. Tim Hardingham, biochemist at the Kennedy Institute, moved to Manchester when the Matrix Centre opened and shifted his research to tissue engineering of cartilage using recombinant DNA (T. Hardingham, interview with author, Wellcome Trust Centre for Cell Matrix Research, University of Manchester, UK, 2008).

31 R. Boot-Handford, interview with author, Wellcome Trust Centre for Cell Matrix Research, University of Manchester, UK, 2008.

32 M. Humphries, interview with author, Wellcome Trust Centre for Cell Matrix Research, University of Manchester, UK, 2008.

33 Manual protocols were also crucial for the introduction of recombinant DNA into the teams working on the haemoglobin gene during the mid and late 1970s (see second half of Section 3). Ramirez, a researcher involved in both haemoglobin and collagen research, initially based his experiments on a Xeroxed copy of the cloning protocols from molecular biologist Tom Maniatis. He referred to these protocols as “the Bible” (F. Ramirez, interview with author, Mount Sinai Medical Centre, New York, US, 2008). Maniatis further published a textbook which became a fundamental source in the circulation of cloning and other recombinant DNA techniques (Maniatis, 1982). On the role of protocols in the circulation of practices and instruments see Lynch, Cole, McNally, & Jordan (2009, pp. 87 and ff.).

34 M. Grant, M. Humphries, K. Kadler, and J. Sheehan (1993b) “Equipment justification” (Michael Grant’s personal archive, Faculty of Life Sciences, University of Manchester, UK, pp. 3–4).

35 M. Dixon, M. Humphries, G. Brady, J. Hewitt, and K. Gull (1995) “An integrated DNA sequencing facility for studies of genome analysis and gene organisation” (Michael Dixon’s personal archive, Faculty of Life Sciences, University of Manchester, UK, pp. 4–8c).

36 M. Dixon, M. Humphries, and K. Gull (1999) “Equipment for semi-automated, fluorescence-based DNA sequencing” (Michael Dixon’s personal archive, Faculty of Life Sciences, University of Manchester, UK, pp. 6–10c).

37 R. Boot-Handford, inverview with author, Wellcome Trust Centre for Cell Matrix Research, University of Manchester, UK, 2008.
